# The Leadership Lab for Women: Advancing and Retaining Women in STEM through Professional Development

**DOI:** 10.3389/fpsyg.2017.02138

**Published:** 2017-12-15

**Authors:** Ellen B. Van Oosten, Kathleen Buse, Diana Bilimoria

**Affiliations:** Weatherhead School of Management, Case Western Reserve University, Cleveland, OH, United States

**Keywords:** women’s leadership development, women in STEM, gender bias, individual change, coaching

## Abstract

Innovative professional development approaches are needed to address the ongoing lack of women leaders in science, technology, engineering, and math (STEM) careers. Developed from the research on women who persist in engineering and computing professions and essential elements of women’s leadership development, the Leadership Lab for Women in STEM Program was launched in 2014. The Leadership Lab was created as a research-based leadership development program, offering 360-degree feedback, coaching, and practical strategies aimed at increasing the advancement and retention of women in the STEM professions. The goal is to provide women with knowledge, tools and a supportive learning environment to help them navigate, achieve, flourish, and catalyze organizational change in male-dominated and technology-driven organizations. This article describes the importance of creating unique development experiences for women in STEM fields, the genesis of the Leadership Lab, the design and content of the program, and the outcomes for the participants.

## Retaining the Best and Brightest

Carrie Jenkins felt the presentation to senior management went really well. As a program manager for a mid-sized manufacturing company, she oversaw the largest engineering project the company had ever had. Senior management recognized her and her team for effectively completing the project on time and under budget and promoted her to senior project manager shortly thereafter. What the management team did not know was that 1 year earlier, she was actively engaged in an external job search.

The vignette above is a true story about Carrie Jenkins^1^, a 44-year-old engineering manager with 20 years of experience. After graduating with undergraduate and graduate degrees in biomedical engineering, she worked as a design engineer for a consumer products company. Outside of work, she was married with three children and struggled to juggle the constant demands of work and family. While she loved her work, her relationship with her manager, Jerry, was a source of constant stress. Jerry had a command-and-control leadership style and preferred to give orders rather than ask for input or engage in dialog. The advice he often gave Carrie was to adopt a more assertive style when presenting to management. He encouraged her to be dominating, such as pounding her fist to emphatically convey important points. Carrie followed his advice and became more authoritative in her style and highly directive with her associates. However, she never felt comfortable, and it wasn’t working well. Her team operated in silos and seemed to wait for her to make all the decisions, which was a constant source of frustration. After years of trying to motivate the team, she concluded that she was not a good fit for the company and began to look elsewhere.

In the midst of her job search, her organization learned of a professional development program specifically for women in STEM fields and invited Carrie to attend. Through the experience, Carrie discovered that her preferred leadership style, which was collaborative and democratic, could work effectively. She developed a personal vision and discovered that her values and strengths overlapped well with the organization’s culture. She gained the confidence to be authentic in her approach. Great things began to happen. Her team trusted her more, became more unified, and performed at a higher level. Carrie became re-engaged and re-energized in her work and suspended her job search. A promotion to Program Manager came a few months after her graduation from the program. Carrie reflected upon her experience in this way: “The biggest eye-opener was that I needed to be true to myself and that I can be effective as myself, but I will never be effective trying to be someone else”.

The Leadership Lab is a professional and leadership development program specifically for women in the science, technology, engineering, and math fields (STEM) just like Carrie Jenkins. In this article, we describe the origin and design of the program and discuss why this type of professional development is needed to advance and retain women in STEM professions. Finally, we highlight initial outcomes of the program.

## Barriers to Advancement

Women’s participation in the United States. labor force has grown exponentially over the past seven decades to the point where women comprised 47% of the workforce and 52% of professional and management occupations in US Bureau of Labor Statistics (2015). However, women continue to be under-represented at the highest levels of leadership with only 5% of Fortune 500 CEOs and 20% of corporate board positions held by women (Catalyst, 2016). In the STEM professions, women represent 46% of scientists, with higher representation in social, biological, and medical sciences, 25% of those in computer and mathematical professions and 12% of those in the engineering professions (US Bureau of Labor Statistics, 2015).

Previous studies have identified obstacles that women must overcome to be successful in the STEM workplace. These barriers and biases have been summarized into the following categories: structural barriers within the educational system; individual and psychological factors; family influences and expectations; and perceptions of the STEM educational and the workplace experiences (Kanny et al., 2014). Despite these barriers to women’s professional achievement, many institutions are focused on increasing the representation and advancement of women in the engineering and computing professions. These include educational institutions (Klawe, 2014; Anderson, 2016), government agencies (National Academies, 2016; National Institutes of Health, 2016), non-profits (Corbett and Hill, 2015; National Center for Women in Technology, 2016; Society of Women Engineers, 2016) and corporations (Google, 2014; Loosvelt, 2016). The primary impetus for this is because an inclusive culture provides tangible benefits for organizations (Manjdo, 2014; Post and Byron, 2015), societies (Katrin Elborgh-Woytek, 2013), as well as for individual women (David Beede, 2011). Despite these benefits, women leave certain STEM professions such as engineering at double the rate of men (Frehill, 2008), creating a brain drain of talent and a persistent challenge for organizations.

Previous organizational efforts to create gender balance in leadership have primarily focused on reducing gender discrimination and increasing attention on diversity and inclusion through company-wide policies and programs, yet such initiatives have failed to create more gender-diverse workforce participation and leadership (Ely et al., 2011). Female STEM professionals continue to experience real barriers to advancement, retention, and leadership. Organizational studies investigating female under-representation, particularly in leadership, have shifted from examining more visible efforts to exclude women (e.g., overt acts of sex-based discrimination such as banning women from the shop floor) to more subtle forces, also known as *implicit bias* or second-generation bias (Ely et al., 2011). Implicit bias consists of stereotypical beliefs about gender that pose invisible barriers to women’s advancement, as well as workplace structures and everyday practices that inadvertently favor men. For example, women often lack access to the informal networks necessary for advancement, potentially disadvantaging them when promotion decisions are being made. Because science and technology professions and industries like science, engineering, and manufacturing are stereotypically viewed as male-dominated, women may experience difficulties in the form of second-generation biases while working in these fields (Heilman et al., 2004).

## Developing Women who Persist and Succeed

In research examining women who persist in engineering careers, individual factors and contextual experiences separated those who remained from those who opted out (Buse et al., 2013; Buse and Bilimoria, 2014). The individual factors included self-efficacy, having a career identity and the ability to see a better future, adaptability, and being engaged at work. Contextual experiences related to a woman’s choice of engineering, work experiences as an engineer, and her family situation. Career commitment was found to be influenced by work engagement, a personal vision, and an interaction between the age and number of children (Buse and Bilimoria, 2014). In other work, researchers examined the organizational conditions under which women are more likely to succeed in STEM workplaces to include supportive managers, policies and practices that encourage work-life integration, and an equitable gender climate (Bilimoria and Lord, 2014). Overall, women who persist understand themselves and what they want from life, nurture supportive relationships personally and professionally, recognize the impact of socio-cultural factors on their ability to achieve, and take initiative to overcome bias and barriers.

Given the barriers, advancing and retaining women in traditionally male-dominated professions requires organizations to implement strategies for professional development tailored to address the gendered context of women’s careers and lives. Research in women’s leadership development suggests a number of factors promote an ascent to leadership, including self-awareness (Taylor et al., 2016), a holistic self-concept and identity (Ely et al., 2011; Debebe et al., 2016; Sugiyama et al., 2016) and a personal vision (Buse and Bilimoria, 2014). Having an understanding of implicit bias including workplace practices is also essential. Women are urged to understand and overcome gender bias (Lanaj and Hollenbeck, 2015); however, many women are not even aware when bias is occurring (Crosby, 1984, 1986).

STEM female professionals benefit from mentors and coaches who provide support and inspiration. Developmental relationships, such as those with mentors and coaches, provide psychosocial support and career development advice (Kram, 1985). Since women learn well through others’ stories of success and struggle (Debebe, 2011), women-only leadership development programs have been recommended (Debebe et al., 2016), especially for populations lacking critical mass such as women in leadership and women in STEM disciplines. Furthermore, peer coaching provides a safe environment for developmental feedback to be exchanged and mutual learning to occur (Parker et al., 2014). In the next section, we explain the participant profile and share program objectives, content, and other design elements of the Leadership Lab.

## Leadership Lab Program Participants and Design

The Leadership Lab Program was developed to address the unique context of women’s experience in technical roles and organizations and to provide participants the knowledge, skills, connections, and support to succeed and catalyze change in their organizations. Research from several streams shaped the design for the program including: why women persist in STEM roles (Buse and Bilimoria, 2014), self-awareness and self-efficacy in women’s leadership development (O’Neil et al., 2015; Sugiyama et al., 2016), emotional intelligence in leadership effectiveness (Goleman et al., 2002), the catalytic power of a personal vision (Smith et al., 2009; Buse and Bilimoria, 2014; Passarelli, 2015) and the positive impact of coaching relationships (Smith et al., 2009). Intentional Change Theory (ICT) provided a framework for participants to engage in professional development. ICT describes a process that facilitates learning, growth, and change through five phases of discovery including the ideal self, the real self, learning agenda, experimentation and practice, and supportive, trusting relationships (Boyatzis and Akrivou, 2006; Boyatzis, 2008).

In the Leadership Lab, five themes characterized participants’ hopes and expectations for their development experience: influencing others in a male-dominated environment, becoming re-energized in their choice of a STEM career, developing “soft” skills including how to communicate with and manage diverse others, and navigating a balance between career and personal life. The Leadership Lab design was purposeful in addressing these components, which are critical for women’s advancement.

**FIGURE 1:**
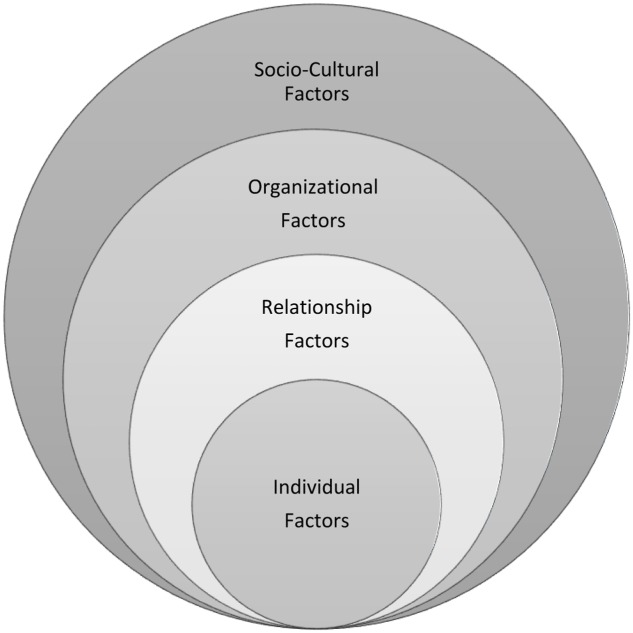
Levels of learning addressed in the Leadership Lab.

Led by female instructors with experience with STEM professions, the program learning objectives included helping participants to (a) understand the complex factors impacting women in male-dominated professions, (b) recognize the value women bring to the STEM workplace, (c) explore factors for leadership effectiveness, and (d) develop strategies and skills to flourish professionally and personally. Individual, relational, organizational, and socio-cultural factors impacting women’s effectiveness and success were explored throughout the program as illustrated in **Figure 1**. An emphasis was placed on helping women to develop self-awareness, self-efficacy, emotional intelligence and coaching capability.

The program design included seven days of experiential learning, scheduled in three modules over three months. The first module focused on the bias, barriers, and opportunities facing women in STEM and the power of a personal vision. The second module addressed skill development in the areas of leadership and emotional intelligence. The third module focused on skills and strategies for leading the way forward. Topics covered during these modules included gender diversity in organizations, implicit bias, resonant leadership, emotional intelligence, self-efficacy, negotiations, leadership presence, and coaching. Participants completed assessments to encourage self-awareness, including a 360-degree report on emotional intelligence. They were paired with an executive coach and met three times over the course of the program. Coaches worked between residencies to help participants explore their passion, values, career aspirations, emotional triggers and developmental focus. Participants also practiced peer coaching in triads which served as a source of ideas, feedback and psychosocial support for team members. Peer coaching triads met three times during the program. On the final day of the program, the manager of each participant was invited to attend a graduation luncheon, where s/he hear a program summary and insights gleaned by the participants.

To date, the program has been offered five times, with three cohort groups representing women in STEM generally and two cohorts representing women in manufacturing. Women participants were nominated and supported by their organizations. Participant work experience ranged from 5 to 25 years. The women represented a variety of positions such as Senior Quality Engineer, Engineering Program Manager and Project Manager for the STEM cohort and Global Environment and Sustainability Manager for the manufacturing cohorts.

## Encouraging Outcomes

Over 50 women have completed the Leadership Lab in the past three years, and the feedback has been overwhelmingly positive. In participant feedback collected post-program through surveys and interviews, four themes were mentioned most by the participants: (a) job promotions, (b) heightened awareness of unconscious bias and how to mitigate it, (c) stories of personal transformation around self-efficacy and breaking counter-productive thinking and behavior, and (d) benefits received from coaching relationships.

Participants have continued to stay connected with their cohort and with the faculty. Each cohort has created a medium to stay connected; several use Facebook groups, one uses a LinkedIn group, and another meets quarterly by video conference. From these connections, we know that the attendees have been retained in their professions, although a long-term study is needed to validate the impact of the program on retention. Based on participant testimonials, it was estimated that about 40% of the women had sought and received a promotion within 12 months of completing the program.

Beyond positioning women for advancement, the program helps women to understand and address the barriers often characteristic of male-dominated workplaces and to know ways to better navigate the environment. Take Andrea, for example. She was a research engineer working for an aerospace organization. After 5 years, she felt stuck and did not feel as if she was going to advance. She applied to the Leadership Lab in hopes it would re-energize her and boost her career prospects. While in the program, Andrea applied for a project manager position, which had been a long-time career ambition and was offered the job. Prior to starting, she was contacted by two male managers at another division who invited her to meet. Andrea looked forward to it, thinking it would be good to get to know her new colleagues before working together. The managers had a different idea. Andrea recalled the experience:

“Their message was, ‘we don’t think you’re the right person for this job. We think our candidate is better and she should have gotten the job.’ They kept pounding me telling me I shouldn’t have been chosen to be project manager. I sat there stunned at first. Then I stopped them and said, “Hold on. I haven’t even started in the role and you are saying that I’m not a good fit for it? I have a PhD in chemistry and am qualified. Before you say that I cannot do this job, let me do it but don’t complain before I even get started. One of them apologized to me a year later.”

Participants report personal transformation especially in terms of deeper self -awareness, social awareness and self-efficacy. In Andrea’s case, she offered:

“The program was an eye opener for me because I felt as if a veil was lifted and I finally saw how under-represented women are in my organization. We have 30 project managers and only 1 woman – me. When I give a presentation, they drill me with more questions. At first, I thought it was me, but my co-workers noticed it too. In meetings, men would take the seats at the table and women would sit behind them. I remember thinking this can’t happen anymore, so at the next meeting, I started sitting at the table. The program gave me courage and tools to manage those difficult situations.

Before the Leadership Lab, I would not have had the guts to tell the two men what I thought as they criticized me for no reason. I would have listened, and then gone to my supervisor and let him deal with it. But I’m different now. I reflect on the situation and first have the difficult conversations before asking others for help. In the past, I would have waited for someone else to tell me about an open position and now I look for them.”

[Research engineer, Aerospace]

An element of the program that participants highly valued was the executive and peer coaching each one experienced. One participant, Trisha, commented:

“For me, this [coaching] was one of the most fulfilling aspects of the course. I learned that I haven’t had much coaching in my career. Receiving feedback from my peers, and a professional coach was awesome! From my peers, I loved having someone listen to me, repeat back what they heard so that I could hear how my words were interpreted, and give me honest feedback. From my coach, I received the greatest gift: someone who helped me articulate my dreams and goals, and bolstered my confidence in being able to achieve them by giving me tools and feedback that helped me realize that what I want is realistic and achievable.”

[Credit and Collections Manager, Manufacturing]

## Concluding Remarks

Innovative professional development approaches are needed to address the ongoing lack of women leaders in science and technology-related fields. This article spotlights a new program designed as a research-based professional development immersion for women. The program aim is to equip women with the capability to navigate the complexities in non-traditional professions. While plans are in place to do more thorough long-term studies, early analysis of the program’s outcomes show increases in participant’s self-awareness, self-efficacy, and ability to persist and excel in their chosen profession. These provide encouraging signs of the intervention’s success in advancing and retaining women in STEM and in the manufacturing professions.

## Author Contributions

EVO was the lead author. KB and DB also made significant contributions to this manuscript.

## Conflict of Interest Statement

The authors declare that the research was conducted in the absence of any commercial or financial relationships that could be construed as a potential conflict of interest.

## Footnotes

1 Names of individuals in this article have been disguised to protect participant identity.
